# Efficacy and Safety of Rituximab in Chinese Children With Refractory Anti-NMDAR Encephalitis

**DOI:** 10.3389/fneur.2020.606923

**Published:** 2020-12-14

**Authors:** Xiangjun Dou, Dongjing Li, Yan Wu, Zhijing Wang, Le Yang, Nan Ma, Dong Wang, Xia Li

**Affiliations:** Department of Pediatric Neurology, Xi'an Children' Hospital, Xi'an, China

**Keywords:** anti-NMDAR encephalitis, rituximab, children, outcome, adverse event

## Abstract

**Purpose:** To assess the efficacy and safety of rituximab treatment as second-line immunotherapy in pediatric cases of anti-NMDA receptor (NMDAR) encephalitis.

**Methods:** We retrospectively recruited 8 patients with anti-NMDAR encephalitis who were treated with rituximab as second-line immunotherapy. We evaluated the clinical features, laboratory examination results and treatment protocols of the Chinese children and defined good outcomes based on the modified Rankin scale (mRS) score (0–2) at the last follow-up.

**Results:** A total of eight pediatric patients (median age 6.7 years; four female) with refractory anti-NMDAR encephalitis were recruited to the study. Rituximab was given after a median duration of disease of 57 days (range 50.5–113.75 days). The use of rituximab led to a significant reduction in the mRS and CD19+ B-cells compared to before rituximab infusion (*P* < 0.05). Five patients (62.5%) had a good outcome (mRS ≤ 2) including four patients (50%) who showed complete recovery (mRS = 0) at the last follow-up. Transient infusion adverse events were recorded in 2 patients (25%). Two patients (25%) had severe infectious adverse events (AEs) and two patients with grade 5 (death). None of the patients developed progressive multifocal leukoencephalopathy (PML).

**Conclusion:** Our study provides evidence that rituximab can efficiently improve the clinical symptoms of anti-NMDAR encephalitis in children. However, due to the risk of adverse infections, rituximab should be restricted in pediatric patients with high rates of mortality and disability.

## Introduction

The common treatment of autoimmune encephalitis includes surgery and first-line immunotherapy consisting of corticosteroids, IVIg or plasma exchange (1–5). It has previously been shown that children are less likely to have detectable tumors and less likely to respond to first-line immune-suppressive agents (1–4). Second-line immunotherapy including cyclophosphamide or rituximab, or in combination is proposed as an effective clinical strategy and can prevent relapse in patients with refractory anti-NMDAR encephalitis (1, 2). Rituximab is an anti-CD20 human chimeric monoclonal antibody that leads to B-cell depletion. Accumulating evidence has demonstrated the benefit of rituximab in children with CNS diseases including anti-NMDAR encephalitis, neuromyelitis optica (NMO) spectrum disorders (NMOSD), and opsoclonus myoclonus ataxia syndrome (OMAS) (2, 6–11). However, serious infectious adverse events (AEs) have been reported in children following rituximab treatment and there remains a lack of comprehensive safety data (6). To increase understanding of the benefits and risks of rituximab usage, we conducted a retrospective study of the efficacy and safety of rituximab usage in Chinese children with refractory anti-NMDAR encephalitis.

## Methods

### Patients

This retrospective study included eight Chinese children from 3 to 12.1 years of age with refractory anti-NMDAR encephalitis. The children were diagnosed in the Department of Neurology at Xi'an Children's Hospital between July 2016 and February 2020. This study was approved by the Ethics Committee of Xi'an Children's Hospital. To evaluate the use, safety, and efficacy of rituximab, we extracted data from patients treated for refractory anti-NMDAR encephalitis that were younger than 18 years. All patients met the following inclusion criteria: (a) patients met the diagnostic criteria for anti-NMDAR encephalitis; (b) patients treated with rituximab after they had failed first-line immunotherapy (methylprednisolone and/or immunoglobulin and/or plasma exchange); (c) patients aged between 0 and 18 years; (d) patients met the definition of refractory anti-NMDAR encephalitis. Refractory anti-NMDAR encephalitis was defined as cases in which there was no clinical improvement or those who had a mRS score of 4 or higher, more than 10 days after the treatment of at least two forms of first-line immunotherapies (1, 9). Patients who were not treated with rituximab or might have had other possible etiologies such as viral encephalitis were excluded from the study.

### Data Collection

Medical information including age, gender, clinical symptoms, diagnosis, laboratory examination results, brain magnetic resonance imaging (MRI) results, electroencephalography (EEG) findings, age at rituximab initiation, the dosage regimen used and the use of other immunotherapies were collected from the medical records or via telephone interviews. Tumor screening (CT of the chest and abdomen, and ultrasound of the pelvic cavity) was performed once each patient was diagnosed with anti-NMDAR encephalitis. All patients were screened for tumors once a year after discharge which included CT and/or ultrasound of the chest, abdomen, and pelvic cavity. The modified Rankin scale (mRS) was used to evaluate clinical disease states and was recorded before and after treatment with rituximab.

### Efficacy and Safety Evaluation of Rituximab

Clinical data including mRS, levels of CD19+ B cells in the peripheral blood and adverse events were collected and used to evaluate the efficacy and safety of rituximab. Good outcomes were considered when patients had a mRS in the range of 0–2, and complete recovery was defined when the mRS was 0 (9, 12). Patients were considered to have relapsed when new symptoms had appeared or when pre-existing symptoms had worsened after the improvement or stabilization of the disorder for at least 2 months (9). The depletion of CD19+ B cells was assessed and defined as <1% of the total lymphocytes in the peripheral blood (13). Infusion adverse events (AEs) including allergic, hypersensitive, or other unwanted effects that occurred during the infusion were also recorded. Any other side effects that may have been attributed to the use of rituximab, in particular, any infectious complications were also recorded. AEs were classified using the Common Terminology Criteria for Adverse Events (CTCAE v5.0) (14).

### Statistical Analysis

Data analysis was performed using GraphPad Prism software, version 5.0 (GraphPad Software, Inc., San Diego, CA). Categorical variables were described using frequency and percentages. Continuous variables those conformance to skew distributions such as mRS, CD19+ B-cell counts and the difference of mRS before and after rituximab were described as median and quartiles and analyzed with a Wilcoxon signed-rank test and a Wilcoxon rank sum test. The differences in the CD19+ B-cell counts before and after rituximab treatment that were normally distributed were analyzed with an independent *t*-test. *P*-values < 0.05 (two-sided) were considered to be statistically significant.

## Results

### Demographics and Clinical Presentation

Data from eight patients (four females) were available. The age at first clinical presentation had a median of 6.7 years (range 3–12.1 years). The first neurological symptoms could be divided into four groups: seizure (four patients, 50%), abnormal behavior and psychosis (two patients, 25%), weakness of the lower limbs (one patient, 12.5%) and aphasia (one patient, 12.5%). During the disease, eight patients (100%) presented with abnormal behavior or psychosis, four patients (50%) showed a decreased level of consciousness, six patients (75%) experienced seizures, eight patients (100%) presented with language and memory deficits, five patients (62.5%) experienced dyskinesia and/or involuntary movements, five patients (50%) showed sleep disorders and three patients (37.5%) presented with breath instability (central hypoventilation) or autonomic dysfunction (urinary incontinence).

MRI results were abnormal in 4 (50%) patients with T2 weighted imaging (T2WI) or fluid-attenuated inversion recovery (FLAIR) hyperintensity signals located in the parietal, occipital and temporal lobes, and the thalamus. The initial EEG showed abnormal results in 7 (87.5%) cases presenting with general or diffuse slowing waves or epileptiform activities. The initial CSF samples were abnormal in 5 (62.5%) cases, with increased white blood cell counts (>5, range 19–115) in 5 (62.5%) and elevated protein levels (>0.45 g/L, range 0.60–0.72) in 3 (37.5%) cases. Anti-NMDAR antibodies were identified in both the serum and CSF obtained from six patients (75%) and CSF only in two patients (25%). The demographic profiles and clinical features of the patients are summarized in Table 1.

**Table 1:** Clinical presentation in patients with NMDAR encephalitis.

**No**.	**Age**	**Sex**	**Initial symptoms**	**Other symptoms during course of the disease**	**Symptom onset until diagnosis (day)**	**Initial MRI**	**Initial EEG**	**Initial CSF**	**Diagnostic marker**
1	5.8 years	Female	Seizures	Language and memory deficits, abnormal behavioral, dyskinesia, involuntary movements, disturbance of consciousness, sleep disorders	19	Increased signal in left temporal and occipital lobe	General slowing and epileptiform activity in left temporal and occipital lobe	20 WBC, 600 mg/dL protein	CSF NMDAR Ab (1:32)and serum NMDAR Ab (1:320)
2	3 years	Male	Seizures, fever	Language and memory deficits, confusion, psychosis, insomnia, dyskinesia and involuntary movements	17	Bilateral increased signal in parietal, temporal and occipital lobe	Diffuse slowing abnormalities	30 WBC, 680 mg/dL protein	CSF NMDAR Ab (1:32)
3	12 years	Male	Weakness of lower limbs	Seizures, psychiatric symptoms, language and memory deficits, abnormal involuntary movements, disturbance of consciousness, breath instability	18	Increased signal in left parietal lobe	Diffuse slowing abnormalities	115 WBC, normal protein	CSF NMDAR Ab (1:32)
4	7.5 years	Female	Aphasia	Seizures, psychosis, memory deficits, dyskinesia, abnormal involuntary movements, disturbance of consciousness, breath instability, insomnia	15	Normal	General slowing	Normal WBC and protein	CSF NMDAR Ab (1:32) and serum NMDAR Ab (1:100)
5	12.1 years	Male	Fever, abnormal behavior, psychosis	Left-sided weakness, language and memory deficits, tremor	20	Normal	Diffuse slowing abnormalities	24 WBC, 720 mg/dL protein	CSF NMDAR Ab (1:32) and serum NMDAR Ab (1:100)
6	5.4 years	Female	Abnormal behavior, psychosis	Psychosis, aggressive behavior language and memory deficits, abnormal involuntary movements, autonomic instability	19	Normal	General slowing and epileptiform activities	Normal WBC and protein	CSF NMDAR Ab (1:32) and serum NMDAR Ab (1:100)
7	10.3 years	Male	Seizures	Psychosis, aggressive behavior, language and memory deficits, insomnia	10	Normal	Normal	19 WBC, normal protein	CSF NMDAR Ab (1:3.2) and serum NMDAR Ab (1:32)
8	4.3 years	Female	Seizures, fever	Psychosis, aggressive behavior language and memory deficits, sleep disorders	17	Increased signal in thalamus	General slowing	Normal WBC and protein	CSF NMDAR Ab (1:3.2) and serum NMDAR Ab (1:320)

### Preceding Therapies

Seven patients had a prolonged course of treatment and two patients (patient 5 and patient 8) had a relapsing course that required multiple immune-suppressive therapies before rituximab treatment. Before the first rituximab infusion, all 8 patients were treated with first-line immunotherapy. Six (75%) patients received corticosteroids (methylprednisolone, 15–30 mg/kg per day for 3–5 days) combined with IVIG (400 mg/kg/d × 5d). One (12.5%) patient (patient 4) received plasma exchange (four cycles) combined with corticosteroid (methylprednisolone, 20 mg/kg/d × 5d) and IVIG (400 mg/kg/d × 5d). One (12.5%) patient (patient 1) received corticosteroid (methylprednisolone, 20 mg/d × 5d), IVIG (400 mg/kg/d × 5d) and cyclophosphamide (600 mg/m^2^ per month, 6 cycles). Six (75%) patients were receiving oral prednisone at the start of rituximab administration, but only 1 patient (patient 6) was receiving ongoing oral steroids with the dose decreased at the last follow-up. The details of the prior therapies received by the patients are summarized in Table 2.

**Table 2:** Treatments and outcome of patients with NMDAR encephalitis before rituximab (RTX).

**No**.	**Tumor**	**Symptom onset until start of immunotherapy, (d)**	**Immunotherapy**	**MRS at diagnosis**	**MRS after aforementioned therapy**	**Treatment response**
1	Negative	20	Steroids, IVIg cyclophosphamide	5	3	Partial
2	Negative	7	Steroids, IVIg	5	4	Partial
3	Negative	14	Steroids, IVIg	5	4	Partial
4	Negative	16	Steroids, IVIg, plasma exchange	5	5	No
5	Negative	21	Steroids, IVIg	3	3	No
6	Negative	18	Steroids, IVIg	3	3	No
7	Negative	10	Steroids, IVIg	3	3	No
8	Negative	18	Steroids, IVIg	4	4	No

### Rituximab Administration

We observed a median time of 16.5 days (range 12.5–89.5 days) to assess the efficacy of prior immunotherapy but no or only partial improvement occurred. Hence, we initiated the regimen of rituximab. The duration of disease before rituximab initiation had a median of 57 days (range 50.5–113.75 days) (Table 3). Two children (patients 4 and 7) received 1,125 mg/m^2^ of rituximab (375 mg/m^2^ × 3) and six children received 1,500 mg/m^2^ (375 mg/m^2^ × 4). One patient (patient 2) began treatment who had ongoing prophylactic antibiotics (cotrimoxazole) at the time of rituximab treatment.

**Table 3:** Rituximab administration.

**No**.	**Disease duration before RTX (d)**	**The duration between initial RTX infusion to last follow-up(m)**	**The interval between first infusion of rituximab and the first sign of clinical improvement (d)**	**Infusion regimen**	**Ongoing treatments at last clinic visit**
1	541	24	18	375 mg/m^2^/ week × 4	None
2	60	27	48	375 mg/m^2^/ week × 4	None
3	50	3.5	32	375 mg/m^2^/ week × 4	None
4	52	12.5	10	375 mg/m^2^/ week × 3	None
5	124	6	No response	375 mg/m^2^/ week × 4	None
6	54	1.7	10	375 mg/m^2^/ week × 4	Oral steroids(tapering)
7	34	2.6	16	375 mg/m^2^/ week × 3	Mycophenolate Mofetil
8	83	37	11	375 mg/m^2^/ week × 4	None

### Efficacy of Rituximab

The median duration between the initial rituximab infusion to the last follow-up was 9.5 months (range 2.825–26.25 months) (Table 3). Significant clinical improvements were observed in seven patients (87.5%) treated with rituximab. The median time between the first infusion of rituximab and the first sign of clinical improvement was 16 days (range 10–32 days). Five patients (62.5%) had a good outcome (mRS ≤ 2) including four patients (50%) who recovered completely (mRS = 0). One (12.5%) patient (patient 5) had no response to rituximab until the last follow-up.

**Figure 1:**
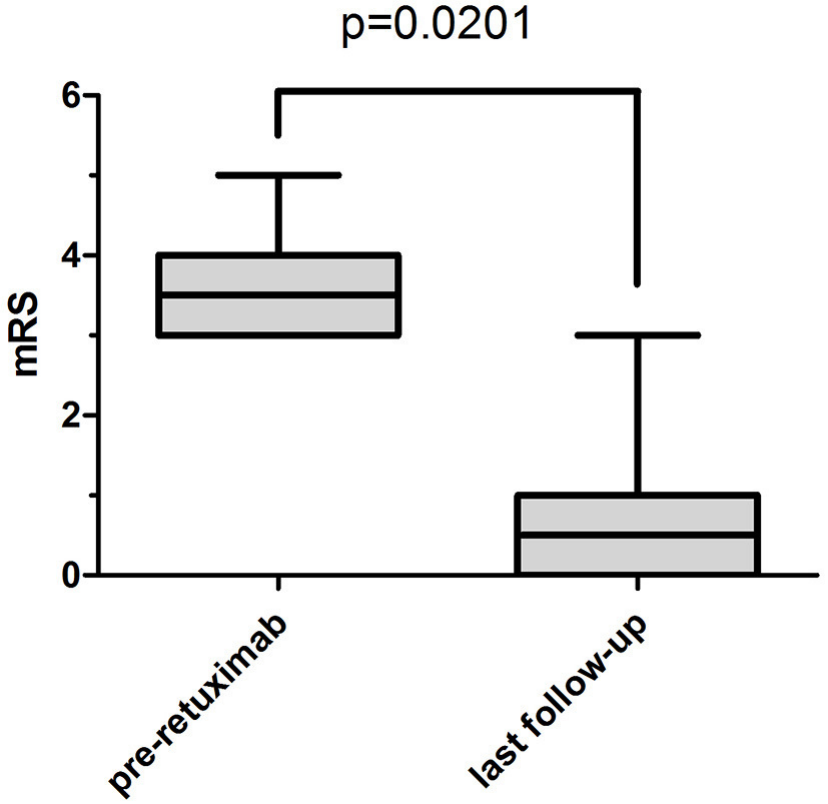
The median and interquartile range of mRS. The median mRS significantly decreased from 3.5 (interquartile range, 3–4) before rituximab treatment to 0.5 (interquartile range, 0–1) at the last follow-up (before patient 3 and patient 6 had severe infectious adverse events) (*P* = 0.0201).

After rituximab treatment, the median mRS at the last follow-up (before infectious adverse events occurred to patients 3 and 6) was 0.5 (range 0–1) compared to 3.5 (range 3–4) before rituximab treatment (*P* < 0.05) (Figure 1). The differences of CD19+ B-cell counts before and at 1 and 4 weeks after rituximab initiation, and the difference of mRS score pre-rituximab and post-rituximab treatment were compared across male and female patients using the *t*-test and Wilcoxon rank sum test. No significant differences were detected in these variables between male and female patients.

**Figure 2:**
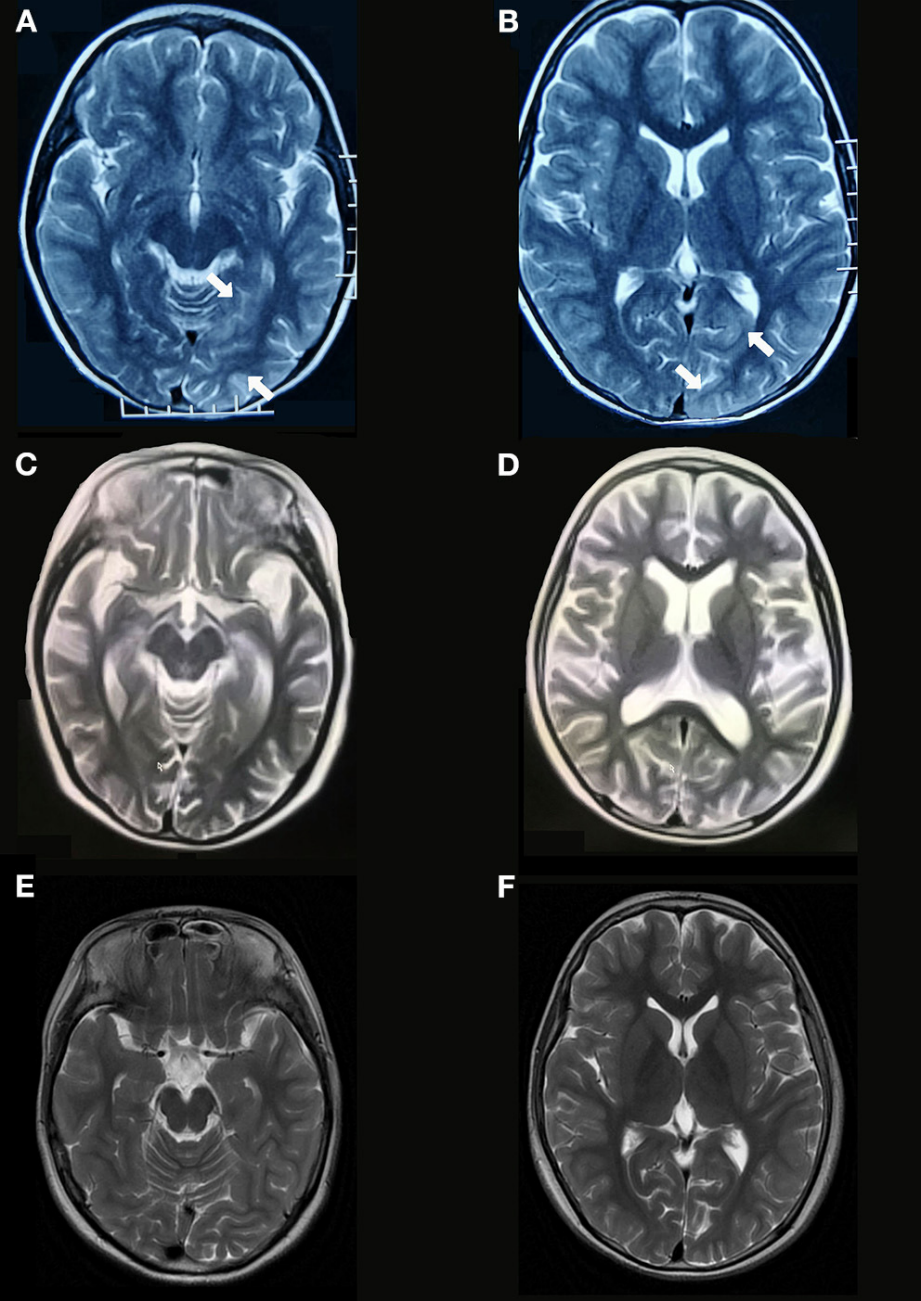
Brain MRI findings of anti-NMDAR encephalitis. Brain MRI obtained 5 days after symptom onset (patient 1, Table 1) shows increased T2 signal abnormalities involving in left temporal and occipital lobes **(A,B)**. Diffuse brain atrophy **(C,D)** are shown on T2 sequences of brain MRI obtained 14 months after symptom onset (before rituximab). Brain MRI at the last follow-up shows normal **(E,F)**.

One female case (patient 1) had a partial response (seizures were controlled and involuntary movements disappeared) to a combination of IVIg and high dose steroids and did not respond to six cycles of cyclophosphamide. However, the girl responded dramatically to rituximab even though the treatment was prescribed 18 months after her initial clinical presentation. The patient's speech significantly improved 18 days after her first infusion of rituximab and her behavioral disorder disappeared gradually after her last infusion of rituximab. She had complete resolution of all symptoms (mRS 0) 2.5 months after her first infusion of rituximab. Her cranial MRI and EEG appeared normal at the last follow-up as compared to the brain atrophy and general slowing waves that were detected before rituximab treatment. The cranial MRI results for this patient are presented in Figure 2).

**Figure 3:**
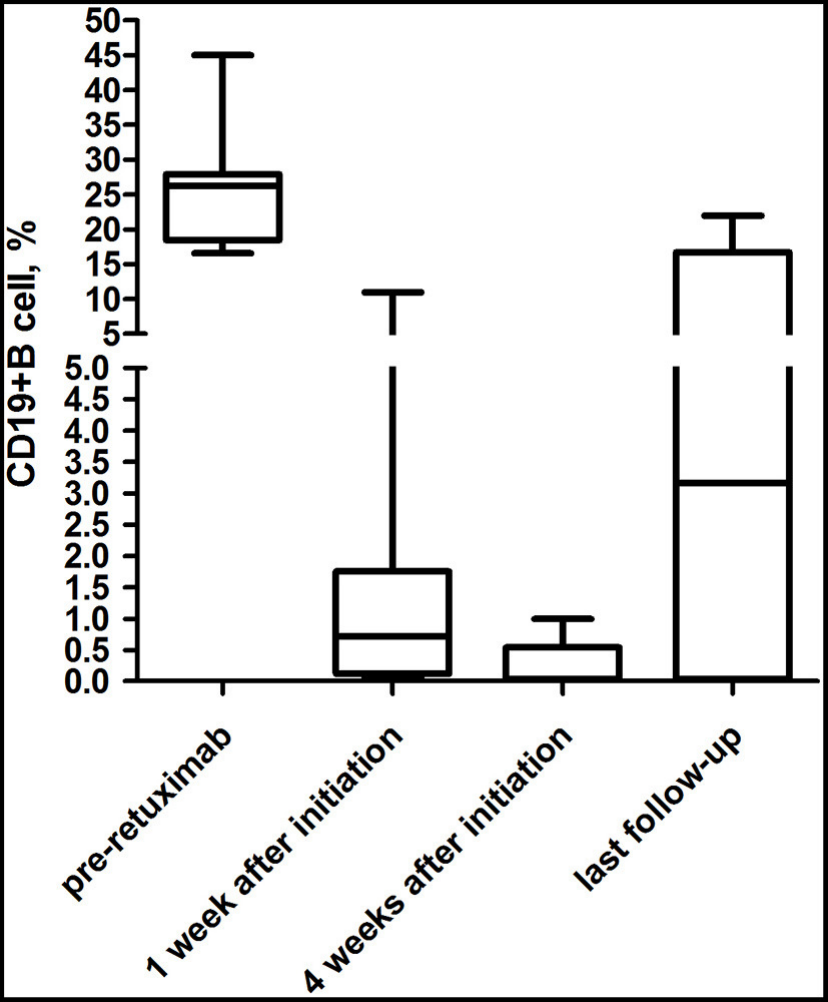
Evolution of CD19+ B-cell counts during the follow-up periods. For most (75%) patients treated with rituximab, CD19+ B-cell depletion occurred rapidly within 1 week after the first infusion of rituximab.

CD19+ B-cell depletion occurred rapidly within 1 week after the first infusion of rituximab in six patients. At 4 weeks after the initiation of rituximab, CD19+ B-cell counts of total lymphocytes in the peripheral blood had a median value of 0.04% (range 0.02–0.5%) compared to 26.2% (range 18.48–27.90%) before the rituximab regimen (*P* < 0.01) (Figure 3). Of the eight patients, 4 (50%) showed an increase in CD19+ B cells that exceeded 1% at a median time of 25.5 months (range 15.4–34.5 months) (Table 4). In the seven patients whose clinical symptoms notably improved, none of them relapsed until the last follow up.

**Table 4:** Comparison between pre- and post-rituximab therapy.

**No**.	**mRS before RTX**	**Best mRS after RTX**	**CD19+ B cell before RTX, (%)**	**CD19+ B cell at 1 week after initial infusion, (%)**	**CD19+ B cell at 4 weeks after initial infusion, (%)**	**CD19+ B cell at the last follow-up, (%)**	**Outcome**
1	3	0	17.00	11.00	1.00	16.00	Complete recovery
2	4	1	27.00	0.50	0.00	22.00	Partial recovery
3	4	1	27.60	0.20	0.05	0.03	Death
4	5	0	28.00	2.00	0.70	6.20	Complete recovery
5	3	3	16.60	0.07	0.03	0.05	No response
6	3	1	45.00	1.00	0.03	0.03	Death
7	3	0	25.40	0.93	0.02	0.13	Complete recovery
8	4	0	22.90	0.10	0.05	17.00	Complete recovery

### Adverse Events (AEs)

#### Infusion Adverse Events

Of the eight patients, two patients (25%) had infusion-related symptom which presented as skin rash during the administration of rituximab. However, the symptoms gradually disappeared after oral cetirizine was given (Table 5). Severe infusion adverse events did not occur in these eight patients.

**Table 5:** Adverse events(AE) of rituximab treatment.

**Adverse events**	**No**.	**Category**	**Details**
Infusion	Patient 3	Grade 2	Skin rash
	Patient 4	Grade 2	Skin rash
Infectious AE	Patient 3	Grade 5(death)	Lung infection (Interstitial pneumonia complicated by respiratory failure). The pathogen could not be found
	Patient 6	Grade 5(death)	Lung infection (*Nocardia farcinica* pneumonia complicated by respiratory failure)

#### Infectious Side Effects

Two patients (25%) had grade 5 (death) infectious adverse events in the follow-up period (Table 5). Infectious AE occurred in patients 3 and 6 at 35 days, and at 104 days after initiation of rituximab, respectively. No cases of PML were reported in the eight patients until the last follow-up.

## Discussion

Dalmau et al. proposed the use of second-line immunotherapy, cyclophosphamide or rituximab or both, in cases that failed to respond to first-line treatments after 10 days. For pediatricians, only one of these second-line agents, rituximab, can be used for those who have failed first-line immunotherapy (1). Rituximab decreased the maturation of B-cells into antibody-secreting cells and also depleted the memory of antibody-secreting B-cells, making it an optimal immunotherapy option for patients with antibody-mediated diseases (1, 2, 11–14).

Currently, rituximab is an off-label immunotherapy that can be used in children with anti-NMDAR encephalitis. This has led to its limited use in severely ill children due to a lack of safety data. In this present study, we described the clinical features, auxiliary examination features, treatment protocols and clinical outcomes of eight Chinese children treated with rituximab. All eight children had no detectable neoplasm supporting previous reports that pediatric patients are less likely to have associated tumors (1, 15–18). Rituximab was administered as a second-line immunosuppressant after a median of 57 days of disease which was longer than other report at 0.1 months (2). The possible explanation is that the treatment was delayed because of the parents of patients having concerns relating to possible side effects or the financial burden of treatment. Various rituximab regimens have been successfully used in pediatric CNS inflammatory disease including 375 mg/m^2^ infused once per week for 3 or 4 weeks, 1,000 mg per week for 2 weeks, and 100 mg weekly for 3 or 4 consecutive weeks (2, 10, 19–21). In the current study, doses of 375 mg/m^2^ once per week for 4 weeks or 375 mg/m^2^ weekly for 3 weeks were given to the 8 patients. All eight children achieved the targeted levels of CD19+ B cell percent ≦1% at 4 weeks after the first infusion of rituximab including the two patients who received 3 doses of rituximab.

In this study, the median duration between the first infusion of rituximab treatment and the first signs of clinical improvement was 16 days, which was similar to another previous report (13). An apparent clinical improvement was found in 87.5% of the patients which was also consistent with other reports (1, 2, 12, 13). Interestingly, despite the duration of disease at rituximab initiation being longer than previously reported, complete recovery was obtained in patient 1 (11, 13, 22, 23). Although previous studies have proposed the early use of rituximab, many Chinese parents refused to use rituximab because of its high cost and risks of side effects (1, 2). Based on the above findings, rituximab remains a potential treatment option for patients with severe disease and are minimally responsive to preceding immunotherapies, even if the duration of disease is very long due to variant reasons.

The CD19+ B-cell levels decreased rapidly within 1 week to levels ≤1% in all eight patients within 4 weeks after the first administration. CD19+ B cells increased to levels >1% in four children whose follow-up periods were longer than 1 year. In a retrospective study of 144 pediatric patients with autoimmune and inflammatory CNS disease treated with rituximab, B cell depletion occurred in 90% of patients and lasted >12 months in 12 patients (2). Data from another retrospective study showed that the proportion of total B cells in lymphocytes was depleted sharply 1 day after treatment and started to regenerate after 3 months (19).

In this present study, no difference in response to rituximab was observed between male and female patients. However, our sample size was small and we will continue to collect more cases to validate these findings. In previous studies, there have been no reports of differences in response to rituximab between male and female patients with anti-NMDAR encephalitis (1–3, 7, 9, 10, 12, 15, 18).

Only one patient received mycophenolate mofetil in our study. None of these children had relapsed after the disease activity stabilized supporting the idea that rituximab might reduce the relapse rate (1). However, a meta-analysis that included a total of 277 patients reported that relapse after the rituximab therapy occurred in 14.2% of patients (24). To prevent clinical relapse, some experts have suggested that reinfusion of rituximab should be implemented after B-cells start to reconstitute or at a regular interval of 6–9 months. Also, other experts have recommended that mycophenolate mofetil or azathioprine is used for at least 1 year after discontinuation of initial immunotherapies (1, 25, 26).

Although antihistamines and corticosteroids were given to all eight patients, infusion-related side effects occurred in two patients. Both patients (patients 3 and 4) had transient infusion-related symptoms and presented with skin rash during the infusion, which has been reported in previous studies (2, 13, 20). According to the literature and our findings, clinicians should pay careful attention to allergic complications during rituximab infusion.

Despite the apparent clinical improvement observed in most patients, after a median follow-up of 9.5 months after rituximab initiation, a significant mortality rate of 25% occurred indicating a major risk of rituximab treatment. In this study, severe infectious adverse events occurred in 25% of the patients which was higher than in previous reports (1, 2, 7, 13, 19) and may be due to several possible reasons. It is possible that multiple confounders were present in our analysis including the small sample size. The guardians of patient 3 stopped treatment as the patient was not responsive to the antibiotics and mechanical ventilation, and had also received corticosteroids during rituximab administration. Also, it is possible that both children received standard dosages of rituximab which were high and typically used to treat large B cell lymphoma.

Several studies have used lower dosages of rituximab in the treatment of autoimmune and inflammatory CNS diseases and showed that the same clinical effects can be achieved without severe adverse events during the infusion and the follow-up period (13, 19–21, 27). We hypothesize that lower doses of rituximab may reduce the incidence of severe adverse events and achieve therapeutic effects. More prospective clinical trials are needed to test this hypothesis. Neither of the patients were commenced on ongoing prophylactic antibiotics. Broncholavage fluid culture of patient 6 in this cohort showed *Nocardia farcinica* which is an opportunistic pathogenic bacteria. Whilst previous study has reported no apparent reduction in serious infections in patients who received antibiotic prophylaxis, our study indicates that antibiotic prophylaxis should be considered in patients who received multiple simultaneous immunosuppressive treatments (2). The final possibility is that both patients received antibiotic treatment and mechanical ventilation in the pediatric intensive care unit, but neither of the patients tried more advanced treatments such as extracorporeal membrane lung treatment (ECMO) due to its high cost and possible side effects. No cases of PML were reported in this cohort, which was similar to previous reports (2, 6, 8, 10). Although the numbers in our study were small and the follow-up periods were short, our data show that PML was rare in children treated with rituximab.

Our study had several limitations. Firstly, the patient number was small and the results might be affected by the retrospective nature of the data extraction. Secondly, our study lacked a control group for comparison. Since this is a very rare and severe disease, only nine patients met our definition of refractory anti-NMDAR encephalitis in the past 4 years. Only one patient who developed refractory anti-NMDAR encephalitis refused to use second-line immunotherapy in which the clinical symptoms showed no improvement and mRS was 3 at the last follow-up (1 year after first-line immunotherapy). All of the guardians of the other eight patients who were enrolled in this study required further treatment and agreed to use rituximab. Because of the medical necessity to respect the families of patients and due to the retrospective nature of the study, we were unable to compare clinical recovery and mortality between the patients. This included those who received rituximab treatment and patients who did not respond to the first-line treatment and were not treated with rituximab.

In future studies, we will continue to collect more cases to better understand how closely linked recovery and mortality is between the rituximab group and other patients who do not receive rituximab. Also, the follow-up periods were relatively short and finally, we did not compare anti-NMDAR antibody titers at times pre- and post-rituximab therapy. Prospective and multicentre studies must be conducted to optimize dosing regimens and for safety monitoring.

## Conclusions

Whilst limited by a small sample size and the retrospective nature of this analysis, our study provides evidence that rituximab could efficiently improve the clinical symptoms in pediatric patients with refractory anti-NMDAR encephalitis. However, due to the risk of adverse infections, rituximab should be restricted to being used in patients with a high risk of disability and mortality.

## Data Availability Statement

The raw data supporting the conclusions of this article will be made available by the authors, without undue reservation.

## Ethics Statement

Written informed consent was obtained from the individual(s), and minor(s)' legal guardian/next of kin, for the publication of any potentially identifiable images or data included in this article.

## Author Contributions

XD wrote the first draft of the manuscript. DL and ZW performed the data analysis. YW, LY, and NM were responsible for data collection. DW and XL initially designed and supervised the study. All authors contributed to the article and approved the submitted version.

## Conflict of Interest

The authors declare that the research was conducted in the absence of any commercial or financial relationships that could be construed as a potential conflict of interest.

## Abbreviations

NMDAR: NMDA receptor
RTX: Rituximab
AE: adverse event
PML: progressive multifocal leukoencephalopathy
CNS: central nervous system
NMO: neuromyelitis optica
NMOSD: neuromyelitis optica spectrum disorders
OMAS: opsoclonus myoclonus ataxia syndrome
MRI: magnetic resonance imaging
EEG: electroencephalography
mRS: modified Rankin scale
CTCAE: Common Terminology Criteria for Adverse Events
CSF: cerebrospinal fluid
T2WI: T2 weighted imaging
FLAIR: fluid-attenuated inversion recovery.
